# Grand challenges in evolutionary and population genetics: the importance of integrating epigenetics, genomics, modeling, and experimentation

**DOI:** 10.3389/fgene.2014.00197

**Published:** 2014-07-08

**Authors:** Samuel A. Cushman

**Affiliations:** United States Forest Service Rocky Mountain Research StationFlagstaff, AZ, USA

**Keywords:** evolutionary and population genetics, epigenetics, bioinformatics, genomics, modeling

## Introduction

**Figure 1:**
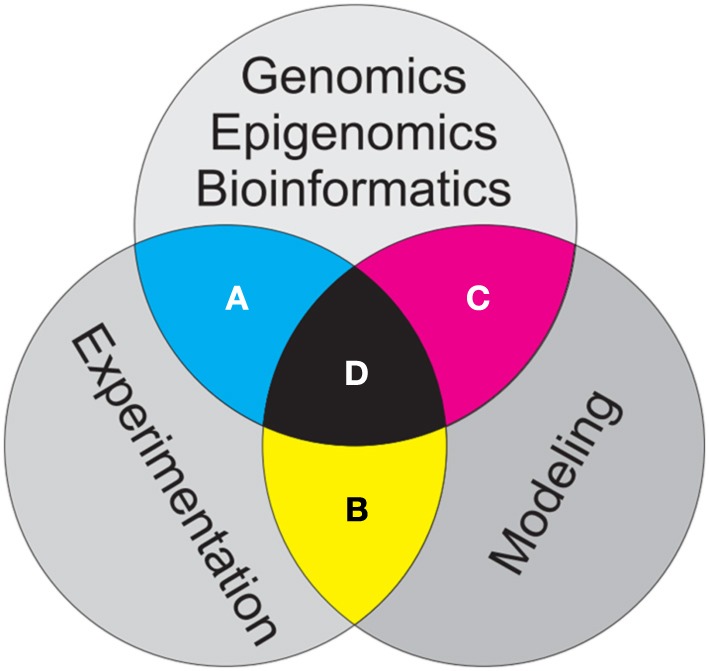
**Schematic showing three major branches of evolutionary and population genetics addressed in this essay**. Bioinformatics work to develop, curate, archive and analyze genomic and epigenomic data, modeling of the influences of population processes on genomic and epigenomic patterns within populations and controlled and replicated experiments to test hypothesized relationships are all critical to advancing our field. The greatest challenges and opportunities lie in the intersections among these three branches of research. **(A)** Bioinformatics should inform design of experiments, and results of genetic experiments should guide what genomic and epigenomic data are collected for a given research effort. **(B)** Modeling should guide design of experiments and generalize and extend experimental results to explore implications of pattern-process relationships across scale, and experiments should guide the parameterization and calibration of models. **(C)** Bioinformatics should provide modelers with genomic and epigenomic data appropriate for model development, calibration, optimization and validation, while models should inform bioinformaticians as to which genomic and epigenomic data is most relevant for a particular question. The three way intersection of bioinformatics, modeling, and experimentation **(D)** provides the strongest potential synergy to advance evolutionary and population genetics.

This is a time of explosive growth in the fields of evolutionary and population genetics, with whole genome sequencing and bioinformatics driving a transformative paradigm shift (Morozova and Marra, 2008). At the same time, advances in epigenetics are thoroughly transforming our understanding of evolutionary processes and their implications for populations, species and communities (Callinan and Feinberg, 2006). These revolutionary changes present tremendous opportunities and challenges to our field (Table 1). In this essay, I will lay out my personal interpretation of what some of the biggest opportunities and challenges are for evolutionary and population genetics over the next decade. I believe that for our field to take full advantage of these tremendous opportunities, we must effectively combine genomics, epigenetics, bioinformatics, experiments and modeling (Figure 1). Genomic pipelines are rapidly producing intractably large volumes of data (e.g., Griffiths-Jones et al., 2008), often without sufficient forethought about what the data will be used for, or how it will be curated, archived, and analyzed. We would be well served by thinking carefully in advance about hypotheses, what data would be best suited to address them, what experiments could be designed to evaluate and validate results, and how powerful modeling approaches could be coupled with experimentation and data mining to generalize experimental results and explore their implications across scales of biological organization from nucleotides to ecosystems.

**Table 1:** **Grand challenges facing evolutionary and population genetics related to genomics, epigenomics, bioinformatics, modeling and experimentation, and their integration**.

**GENOMIC, EPIGENOMIC, AND BIOINFORMATICS GRAND CHALLENGES**	
A	Improved efficiency and effectiveness of whole genome sequencing and development of broad libraries of genomes of non-model organisms.
B	Improvements of fine-scale genetic mapping to quantify patterns of genetic linkage across genomes.
C	Improved quantification, measurement, and understanding of the genetic architecture and processes controlling heterosis, epistasis and pleiotropy, and other interactions between loci and alleles within the genome.
D	Improved understanding of the architecture and processes affecting heritable variation in gene activity not caused by changes in DNA sequence.
E	Understanding the architecture and processes driving changes of transcriptional potential of a cell.
F	Improved understanding of causes and consequences of DNA methylation and histone modification.
G	Improved understanding of interactions between genomic variation and epigenetic processes, such as effects and heritability of repressor proteins attached to silencer regions of DNA.
H	Improved methods and tools for organizing, analyzing, storing and retrieving vast genomic, and epigenomic datasets.
**MODELING GRAND CHALLENGES**	
A	Developing computationally efficient spatially explicit, individual based models that simulate dispersal, mating, genetic exchange, and mortality as functions of cost-distance between individuals resulting from differential patterns of movement in heterogeneous landscapes.
B	Incorporating selection into spatially explicit, individual based genetics models, such that models allow evaluation of differential patterns of selection across complex fitness landscapes, and the interaction of differential patterns of gene flow with differential patterns of local selection.
C	Improving how genomic data are modeled in individual-based, spatially explicit gene flow and selection models.
D	Using the improved models described in (A–C) to evaluate relationships between landscape resistance, landscape heterogeneity, population distribution and density and spatial patterns of allelic richness, heterozyosity, inbreeding coefficient, and effective population size.
E	Using the improved models described in (A–C) to evaluate time lags in the emergence of genetic structure and equilibration of genetic diversity in spatially structured populations.
F	Using the improved models described in (A–C) to evaluate mechanisms for sympatric and peripatric speciation as functions of restricted gene flow and differential local directional selection.
G	Using the improved models described in (A–C) to evaluate the interactive effects of landscape heterogeneity, landscape dynamics, and population dynamics on power of different statistical modeling approaches to reliably detect and predict changes in genetic diversity, population structure, and fitness in response to spatial patterns in the environment and fluctuations in population size and environmental conditions.
**EXPERIMENTATION GRAND CHALLENGES**	
A	Designing and implementing replicated common garden experiments in which genotypes collected from across broad environmental gradients are reciprocally transplanted in replicated experimental gardens that span the range of environmental conditions in the field.
B	Incorporating multi-species, community-genetics designs into replicated common gardens to evaluate the interactions between genetic characteristics of foundation species and the genetic characteristics and composition of associated communities.
C	Conducting long-term experiments in which strength of selection is controlled to identify the genomic and epigenomic structure and processes underlying adaptation.
D	Conducting long-term experiments in which rates of migration and strength of selection are controlled in a spatially structured environment to quantify interactions between gene flow, epigenetic processes and selection in influencing genetic diversity, fitness and reproductive isolation.
E	Conducting long-term experiments in which species interactions, such as competition, commensalism and predation, are manipulated across gradients of differential gene flow and selection to understand how population process across multi-species communities interact to drive evolution of the individual species.
**GRAND CHALLENGES INVOLVING THE COMBINATION OF MODELING WITH GENOMICS/EPIGENOMICS/BIOINFORMATICS**	
A	Combining simulation modeling and genomic data to better understand processes of non-additive gene interaction, such as epistasis and polygenic effects on phenotype, and how they influence evolution across complex spatially heterogeneous adaptive landscapes.
B	Combining simulation modeling and genomic/epigenomic data to better understand the causes and consequences of pleiotropy in natural and simulated populations, specifically how spatial and temporal fluctuations in heterogenous adaptive landscapes may affect the outcome of fitness tradeoffs of pleiotropic effects in terms of patterns of gene frequency across a spatially structured population.
C	Using simulation modeling to evaluate the evolutionary influences of epigenomic processes, such as DNA methylation, histone modification and repressor proteins, in spatially complex and temporally varying environments and in multi-species interactions.
D	Using improved understanding of genomic and epigenomic architecture to improve realism and usefulness of spatially explicit, individual-based simulation models.
E	Using simulation models to evaluate what kinds of genomic and epigenomic data to produce for a given research objective, in terms of what kinds of markers, how many markers, from what parts of the genome, from how many individuals, and from which locations across the population.
**GRAND CHALLENGES INVOLVING THE COMBINATION OF EXPERIMENTATION WITH GENOMICS/EPIGENOMICS/BIOINFORMATICS**	
A	Designing controlled and replicated experiments to test hypotheses about gene linkage, epistasis, pleiotropy, and polygenic effects on fitness.
B	Designing controlled and replicated experiments to test hypotheses about heritable variation gene activity that is not caused by changes in DNA sequence.
C	Designing controlled and replicated experiments that are able to separate and quantify the relative effects and interactions of genomic and epigenomic processes in driving evolution.
D	Using information from whole genome scans and gene mapping to inform experiments as to what loci and what markers to include as response factors in experiments that manipulate selection gradients and species interactions.
**GRAND CHALLENGES INVOLVING THE COMBINATION OF MODELING AND EXPERIMENTATION**	
A	Using simulation modeling to evaluate alternative experimental designs in terms of tradeoffs in sample size, experimental complexity, variance and effect sizes to inform design of optimal experiments.
B	Using experiments to confirm and validate predictions of simulation modeling.
C	Using simulation modeling to generalize experimental findings by evaluating potential outcomes of identified processes in novel conditions, heterogeneous landscapes, fluctuating environments, and across broad ranges of spatial and temporal scale.
**GRAND CHALLENGES INVOLVING THE COMBINATION OF MODELING, EXPERIMENTATION, AND GENOMICS/EPIGENOMICS/BIOINFORMATICS**	
A	The greatest opportunities for advancing the fields of evolutionary and population genetics involve combining modeling, experimentation, genomics, epigenomics, and bioinformatics.
B	Combining bioinformatics with modeling and experimentation to link vast genomic and epigenomic databases to spatially explicit simulations which are then validated and calibrated by controlled and replicated manipulative experiments.
C	Experiments provide decisive proof of cause-effect relationships relating genomic and epigenomic variation to evolutionary and population genetic processes, while modeling allows exploration and generalization of the implications of these relationships across scales in spatially complex and temporally varying conditions, such as predominate in actual populations.

Today our field is justifiably obsessed with the explosive emergence of vast genomic data sets and the opportunities of integrating epigenetics with genomics to explore epigenomic patterns of gene regulation (Griffiths-Jones et al., 2008; Suzuki and Bird, 2008). However, the informatics challenges that attend this emergence are often not fully appreciated. The sheer vastness of genomic data sets is spectacular to contemplate, and can be terrifying to witness. Scientists are often overwhelmed and drown in the vastness of these emerging data. More data does not necessarily lead to better understanding. Often the flood of data can force scientists to focus on data storage and curation to such a degree that they completely lose sight of hypotheses about relationships between these data and biological process, how the patterns we observe in these vast data sets can be tested through controlled and replicated experimentation, and how the results can be generalized across scale through simulation and modeling.

## Intersection of genomics and epigenetics with experimentation

### Data without experiments and experiments without appropriate data are both equivocal

Experimentation has always been one of the most effective means to achieve reliable knowledge (Wright, 1984). Through replication and control, variation can be quantified and accounted for and spurious effects can be removed, leading to reliable inferences about drivers and strong tests of hypotheses. In our field, linking common garden experiments (Whitham et al., 2006) with genomic and epigenomic datasets presents a tremendous opportunity to advance understanding of genetic controls on phenotype and fitness (Figure 1A). In the sea of genomic data, it is all too tempting to use data mining techniques to seek correlations between genetic patterns and some process of interest. Finding such correlations suggests hypotheses, but does not provide a strong basis to evaluate whether these hypotheses may be true. The phenomenon of the under-determination of theories by facts suggests that there may be innumerable ways in which a given observed pattern of genetic variation could have been produced in a population, and to avoid logical inferential errors of affirming the consequent it is absolutely essential to test hypothesis in controlled and replicated experiments, such as common gardens.

## Intersection of experimentation and modeling

### Experiments without models are not extensible; models without experiments are not verifiable

Experiments provide a powerful means to control one or a few processes hypothesized to drive genetic and epigenetic structure of populations (e.g., Kohler, 1994). However, experiments necessarily are limited to a few interactions, at relatively small scales and over relatively short temporal extents. Simply put, experiments without models are not extensible, and models without experiments are not verifiable (Figure 1B). Simulation modeling provides tremendous abilities to explore the implications of alternative hypotheses (Epperson et al., 2010; Landguth and Cushman, 2010). This allows researchers to a priori evaluate sampling designs and analytical approaches to optimize the research to provide high power to evaluate hypotheses. In addition, simulation enables generalization of the results of experiments across scales to explore the implications of causal relationships in real populations. Ecosystems are the stage on which the play of evolution is acted, and ecosystems are complex, spatially structured, and temporally varying. Our hypotheses typically focus on relatively simple relationships between mechanisms and responses, and our experiments to test them focus on these relationships at small scales over short time periods. Simulation modeling is critical to explore how these pattern-process relationships propagate across scale and how variation in these processes across space and through time influences their outcomes.

## Intersection of genomics and epigenetics with modeling

### Models without data are not compelling; data without models are not informative

Integration of genomic and epigenomic datasets with genetic modeling is improving model testing, validation and calibration (Figure 1C). Spatially explicit, individual based simulation modeling has advanced such that relationships between environmental characteristics, population structure and the genetic or epigenetic characteristics populations can be rigorously modeled (Landguth and Cushman, 2010; Landguth et al., 2012). The genomic revolution is providing researchers vast data sets comprising the genomic and epigenomic characteristics of many individuals which enables models to be optimized to training datasets, and validated using independent testing data. Just as models without data are not compelling, data without models are not informative. Simulation modeling has tremendous potential to quantify genetic processes, explore how they propagate across space and through time, and predict the effects of changes to the pattern-process relationship.

## Putting it all together

In the sections above I described the challenges and opportunities presented by the intersection of vast genomic and epigenomic datasets, controlled genetic experiments, and simulation modeling. There is a synergistic dependence among these three fields in advancing evolutionary and population genomics. Data alone are not informative. Models alone are not compelling. Experiments alone are not generalizable. It is the intersection among these three different scientific endeavors that provides the best means of addressing the most difficult and important challenges in evolutionary and population genetics.

Perhaps the best way to illustrate this synergy is through an example of how the three-way intersection of genomic data, controlled experiments and simulation modeling might be implemented (Figure 1D). For the sake of illustration, our task is to predict the effects of climate change on gene flow and adaptive evolution of a population across a large geographical extent. We hypothesize that gene flow of this species would be affected by distance among individuals, and that the population would be differentially adapted to environmental conditions across the range (e.g., Landguth et al., 2012). With this hypothesis we might begin by conducting a large simulation experiment to evaluate how different degrees of gene flow and different strengths of selection across environmental gradients would be expected to affect genetic characteristics of the population. The results of these simulations would guide sampling design to detect the hypothesized relationship across a reasonable range of effect size and variability. We would implement this sampling regime, producing a large spatially referenced genomic or epigenomic data set.

Next, we would calibrate, optimize and validate models predicting the population process from the pattern in the observed genomic or epigenomic data (e.g., Cushman et al., 2006). We then would use the simulations previously conducted to evaluate how well the population process inferred from the empirical data optimization matches the patterns produced through simulation of the same processes (e.g., Shirk et al., 2012). This is a different example of synergy between modeling and genomic data, one in which the data are used to infer a population process and simulations are used to evaluate how well that inferred process can explain the observed data. This combination of empirical modeling and simulation modeling would identify the most supported candidate models explaining influences of gene flow and selection on the genetic characteristics of the population.

A controlled and replicated experiment would then be designed to evaluate the working hypothesis developed through empirical analysis and simulation (e.g., Whitham et al., 2006). This would involve synergy between genomics and experimentation and between modeling and experimentation. First, we would design the experiment to control the factors identified through the simulation modeling and empirical optimization to be the putative drivers of observed patterns of genetic variation. For example, if we found one pattern of genomic or epigenomic variation was associated with warm and dry climatic conditions, while another was associated with cold and wet conditions, we could construct a network of experimental common gardens replicated across the climate gradient. We could use simulation modeling to evaluate how many individuals would be needed in each garden and how many gardens would be needed to provide high power to detect the inferred fitness relationship, if present. There would also be synergy between the genomic data and experimental design; we would reciprocally grow the genotypes that evinced the nonrandom patterns of apparent gene flow and selection. This is an example of synergy between genomic sampling and experimental design, in which patterns of genomic data across the putative selection gradients are then used to select individuals expressing those genetic characteristics for reciprocal transplanting across the experimental garden network. Designing the experimental garden to replicate and control the factors identified by modeling as likely drivers of the genetic patterns in the population, and reciprocally transplanting in all gardens the genotypes which were found to be non-randomly associated with the putative selection gradients, would provide a rigorous basis of evaluating whether the processes inferred from empirical optimization and simulation actually are responsible for the observed genomic or epigenomic structure of the population.

Suppose the reciprocally transplanted common garden experiment confirmed the hypothesized relationship between climate and the pattern of genomic or epigenomic variation across the population. We would then explore the implications of the identified process through further simulation modeling. We might want to simulate what the expected genetic structure and allele frequency would be, given the process, in different study areas than were used to build the original model. We then could test the model further by sampling additional genomic samples from this new study area and evaluating how well the observed genetic structure and allele frequencies match that predicted by simulations based on the process identified in the experiments. This is an example of a three way synergy between genomic data, modeling and experimentation.

## Conclusion

Perhaps the greatest challenge facing the fields of evolutionary and population genetics today is to produce, process, curate, archive and analyze immense genomic datasets in a way such that research is led by a priori hypotheses, integrated with powerful modeling, and, where possible, linked to replicated and controlled experiments to test putative relationships between population processes and evolutionary and population genetic responses. No single person has the expertise or the time to effectively bring these components together. More than ever, success in advancing our field will depend on collaborations across large multi-disciplinary groups. Experts in the development of genomic and epigenomic data are needed to produce the raw genomic data for subsequent analysis. Bioinformatics specialists are needed to provide programming and computer science expertise to efficiently process, curate, archive and analyze vast genomic datasets, and to effectively utilize high performance computing resources. Modelers will be needed to work with the bioinformaticians to explore the implications of hypotheses a priori, to refine hypotheses by optimizing fit to observed data, and predict how observed pattern process relationships may propagate across scale through space and time. Experimenters should work closely with modelers to rigorously test hypotheses in controlled and replicated experiments. To be successful this entire integration should be led by theoreticians who have a coherent vision for how each of these parts will synergize to address focused and falsifiable questions of importance in advancing the field.

### Conflict of interest statement

The author declares that the research was conducted in the absence of any commercial or financial relationships that could be construed as a potential conflict of interest.
